# Prevalence and covid 19 vaccination rate in a population of patients with schizophrenia and a substance use disorder

**DOI:** 10.1192/j.eurpsy.2022.685

**Published:** 2022-09-01

**Authors:** S.L. Romero Guillena, I. De Burgos Berdud, R. Gálvez Medina, E. Serrano Rodriguez

**Affiliations:** UGC Salud Mental Virgen Macarena,, Psychiatry, seville, Spain

**Keywords:** schizophrénia, vaccination rate, covid 19, Prevalence

## Abstract

**Introduction:**

As of August 27th, 2021, the diagnosed cases of COVID 19 in Spain are 4 758 003 with a prevalence of 10.03%. 68.4% of the Spanish population is fully vaccinated

**Objectives:**

Primary: To compare the prevalence of COVID infection in a cohort of patients with schizophrenia to patients with a dual diagnosis of schizophrenia and substance use disorder Secondary: To compare the rate of fully vaccinated patients diagnosed with schizophrenia with and without a coexisting substance use disorder.

**Methods:**

Retrospective descriptive study. The population in study is made up of patients with schizophrenia (46) and dual diagnosis schizophrenia (28) (following DSM 5 criteria) Confirmed cases were those cases with positive PCR

**Results:**

There was not a stadistically significant difference in the prevalence of COVID 19 infection between both groups of patients. The prevalence of COVID infection among the dual diagnosis schizophrenia was 3.57% compared to 6.5% in those without coexisting substance abuse disorder. Relative to vaccination rate, we didn’t find a stadistically significant difference between both groups. However, there was a higher vaccination rate in the dual diagnosis schizophrenia group (82.12%) compared to the non-dual diagnosis schizophrenia group (69.56%)

**Conclusions:**

The prevalence of COVID 19 infection in the dual diagnosis schizophrenia cohort is 3.57% and in the group of patients with schizophrenia without substance abuse disorder is 6,5%. In those with dual diagnosis schizophrenia the vaccination rate was un 82.12%. It was 69.56% in those without coexisting substance abuse disorder.

**Disclosure:**

No significant relationships.

